# Dynamic changes in diffusion measures improve sensitivity in identifying patients with mild traumatic brain injury

**DOI:** 10.1371/journal.pone.0178360

**Published:** 2017-06-12

**Authors:** Alexander W. Thomas, Richard Watts, Christopher G. Filippi, Joshua P. Nickerson, Trevor Andrews, Gregory Lieberman, Magdalena R. Naylor, Margaret J. Eppstein, Kalev Freeman

**Affiliations:** 1 Department of Surgery, University of Vermont, Burlington, Vermont, United States of America; 2 Department of Radiology, University of Vermont, Burlington, Vermont, United States of America; 3 Department of Neurology, University of Vermont, Burlington, Vermont, United States of America; 4 Hofstra North Shore LIJ School of Medicine; Hempstead, New York, United States of America; 5 Philips HealthTech, Cleveland, Ohio, United States of America; 6 Department of Psychiatry, University of Vermont, Burlington, Vermont, United States of America; 7 U.S. Army Research Laboratory, Human Research and Engineering Directorate, Aberdeen Proving Ground, Aberdeen, Maryland, United States of America; 8 Department of Computer Science, University of Vermont, Burlington, Vermont, United States of America; University of Minnesota, UNITED STATES

## Abstract

The goal of this study was to investigate patterns of axonal injury in the first week after mild traumatic brain injury (mTBI). We performed a prospective cohort study of 20 patients presenting to the emergency department with mTBI, using 3.0T diffusion tensor MRI immediately after injury and again at 1 week post-injury. Corresponding data were acquired from 16 controls over a similar time interval. Fractional anisotropy (FA) and other diffusion measures were calculated from 11 *a priori* selected axon tracts at each time-point, and the change across time in each region was quantified for each subject. Clinical outcomes were determined by standardized neurocognitive assessment. We found that mTBI subjects were significantly more likely to have changes in FA in those 11 regions of interest across the one week time period, compared to control subjects whose FA measurements were stable across time. Longitudinal imaging was more sensitive to these subtle changes in white matter integrity than cross-sectional assessments at either of two time points, alone. Analyzing the sources of variance in our control population, we show that this increased sensitivity is likely due to the smaller within-subject variability obtained by longitudinal analysis with each subject as their own control. This is in contrast to the larger between-subject variability obtained by cross-sectional analysis of each individual subject to normalized data from a control group. We also demonstrated that inclusion of all *a priori* ROIs in an analytic model as opposed to measuring individual ROIs improves detection of white matter changes by overcoming issues of injury heterogeneity. Finally, we employed genetic programming (a bio-inspired computational method for model estimation) to demonstrate that longitudinal changes in FA have utility in predicting the symptomatology of patients with mTBI. We conclude concussive brain injury caused acute, measurable changes in the FA of white matter tracts consistent with evolving axonal injury and/or edema, which may contribute to post-concussive symptoms.

## Introduction

Traumatic brain injury (TBI) is a significant medical problem worldwide. In the United States, visits to emergency departments (ED) for TBI increased more than 8-fold compared to the total increase in ED visits between 2006–2010, likely reflecting a combination of increased TBI exposure, awareness, and diagnosis [1]. Of all ED visits for concussions or TBI, approximately 75% of individuals are treated and released with diagnoses of mild TBI (mTBI) [2]. mTBI frequently results in cognitive deficits, motor dysfunction and emotional dysregulation [3]. Severity of axonal injury is a determinant of recovery following severe TBI [4], and diffusion tensor imaging (DTI) has emerged as an imaging modality for mTBI that can quantify white matter damage using DTI metrics such as fractional anisotropy (FA), radial, axial, and mean diffusivity (RD, AD, MD) [5–7]. However, both increases and decreases in FA may occur at different time points after TBI, and this, coupled with normal variability in DTI metrics in the population at large, represents a substantial challenge for the diagnostic use of DTI in mTBI [8–11].

If large enough, studies comparing mTBI to control subjects may average out between-subject variability in DTI metrics and show group differences, but natural variation in DTI measures limits the diagnostic value for detecting relatively subtle effects of mTBI in individual patients [12, 13]. Animal studies suggest that reductions in FA occur late (7 days) after injury and not in the first 24 hours [14]. A recent meta-analysis of human subjects suggested that increases in FA occur early and drops in FA take longer to evolve, but this observation is based on a composite analysis of multiple separate studies [15]. There are not yet studies of longitudinal studies of DTI metrics at multiple time points during the first week after injury.

We hypothesized that longitudinal imaging at multiple times during the first week after concussion would overcome the limitation of anatomic and mechanistic heterogeneity, and provide increased sensitivity for detection of white matter injuries. The purpose of this study was to quantify the acute changes that occur in DTI metrics in human mTBI subjects across the first week of injury. In addition, we sought to test the hypothesis that acquiring DTI data from mTBI patients at two time points within the first week of injury could improve identification of mTBI subjects compared to controls and delineate injury in mTBI subjects when focusing on the axonal regions of interest most commonly reported as abnormal in the mTBI-DTI literature [16]. The utility of genetic programming in the analysis of DTI metrics was explored as a novel way to diagnose patients who have suffered a mTBI and to predict future clinical outcome.

## Materials and methods

### Study design and setting

This was an Institutional Review Board approved study. All participants provided written informed consent prior to participation in this study and no verbal consent was obtained.

We performed a prospective, controlled cohort study of adult patients with mTBI patients and healthy controls recruited from the emergency department at a single tertiary care academic medical center. Controls included both trauma patients with isolated extremity injuries without head trauma, as well as healthy, normal subjects. MRI of the brain was performed within 3 days of injury and again between 5 and 10 days post-injury, with a target interval of 7 days.

### Study population

Between June 2011 and December 2012 patients coming to the emergency department were screened for eligibility by research staff. Eligible patients were those aged 18–60 years old who were diagnosed with mTBI, defined as an isolated head injury with an injury severity score (ISS) for any other organ system <2 and with two or more concussive symptoms including headache, loss of consciousness, blurred vision, confusion, dizziness, memory problems or poor balance. Patients were excluded if they: 1) did not have two or more concussive symptoms; 2) had severe TBI or past history of severe TBI (i.e., requiring surgery or rehabilitation); 3) were unable to complete initial MRI within 72 hours of injury; 4) had a pre-existing neurological disorder; 5) had a psychiatric condition (including depression or anxiety) requiring medical treatment within the past year; 6) had a history of substance abuse; 7) had any contraindications to MRI scanning. The control group was comprised of normal volunteers without acute injury who responded to flier advertisements and non-head injured extremity trauma patients who presented to the emergency department without head trauma or TBI-associated symptoms. All controls were subject to the same exclusion criteria as mTBI patients, aside from those pertaining to acute head injury.

### Clinical data collection

Initial clinical data were prospectively extracted in the emergency department by research staff, through discussion with the patients’ healthcare providers, structured patient evaluations and interviews, and reviews of medical records. Additional research-specific details were also collected from questionnaires and the Immediate Post-Concussion Assessment and Cognitive Testing (ImPACT) testing battery [17]. Follow-up data collection was performed by research staff at the time of repeat MRI. All study data were double-entered and compared for accuracy using the Research Electronic Data Capture (REDCap) tools hosted by UVM [18]

### MRI acquisition

Brain MRI was performed at the earliest possible time point after injury and again approximately one week later. All initial scans were completed <72hrs after injury and the period of 7–10 days following injury for the follow up scan was selected in order to cover the time period when most patients are maximally symptomatic after concussion [19]. MRI data were acquired on a Philips Achieva TX 3.0 Tesla (Philips Healthcare, Best, Netherlands) MRI scanner using an 8-channel brain coil with dual quasar gradients (maximum gradient strength 80 mT/m, slew rate 100 T/m/s). T1-weighted images were acquired using a 3D inversion recovery spoiled gradient echo technique (TE/TR/TI/flip angle = 3.7ms/8.1ms/1008ms/8° with a SENSE factor of 1.5). A sagittal acquisition matrix of 240x240x160 provided whole-brain coverage with an isotropic 1mm spatial resolution and a scan time of less than 8 minutes. Diffusion-weighted images were acquired using a single-shot, spin-echo EPI acquisition with b = 1000 s/mm^2^ with 46 uniformly distributed, non-collinear directions. An additional 6 images were acquired with no diffusion weighting (b = 0 s/mm^2^). The acquisition matrix was 120x120 with a field of view of 240x240mm^2^ using a SENSE factor of 2. 59 contiguous 2mm-thick slices were acquired, aligned AC-PC. TE/TR = 68ms/10000ms with a scan time of 9 minutes.

### Outcome measures

Following the first MRI, research staff administered the ImPACT neurocognitive testing battery version 2.0. The ImPACT test measures attention span, working memory, sustained and selective attention, response variability, non-verbal problem solving, and reaction time; each of which is sensitive to mild cognitive impairment. ImPACT also includes a 22-item symptom score based on patient self-rating on a Likert scale ranging from 0 (the symptom was not experienced at all) to 6 (the symptom was the worst they had ever experienced). The 22 ImPACT symptoms include: headache, nausea, vomiting, balance problems, dizziness, fatigue, trouble falling, sleeping more than usual, drowsiness, sensitivity to light, sensitivity to noise, feeling dazed or stunned, irritability, sadness, nervousness, feeling more emotional than normal, numbness or tingling, feeling slowed down, feeling mentally foggy, difficulty concentrating, difficulty remembering, and visual problems. Repeat ImPACT testing was done at 7–10 days following injury to determine the primary clinical outcome of the total number of post-concussive symptoms (ranging from 1–22). All ImPACT tests were administered in a quiet conference room directly outside the emergency department.

### DTI data processing

The diffusion-weighted volumes were manually examined by a single research associate to ensure fit for artifacts due to, for example, cardiac pulsatility and subject motion. Corrupted volumes were excluded from the subsequent analysis or subjects were removed from analysis if more than 4 of the 46 volumes required exclusion. The remaining volumes were coregistered using an affine transformation to correct for both head motion and eddy current-induced distortions. The data were then fit to the diffusion tensor model using FSL4 [20] to generate FA maps. FA maps were spatially normalized using the tract-based spatial statistics [20] (TBSS) processing stream built into FSL, see supplemental methods for the step-by-step processing stream. FA maps were transformed and resampled to 1mm isotropic resolution in the template MNI 152 space. Eleven *a priori* regions of interest (ROIs) were selected as those most commonly reported in the mTBI-DTI literature [16]: the splenium, body and genu of the corpus callosum (CC); and the left and right posterior limbs of the internal capsule (PLIC), uncinate fasciculus (UF), corona radiata (CR), and corticospinal tract (CST). ROIs were pre-defined in MNI 152 space using the Johns Hopkins white matter anatomical atlas [21–23], and were of uniform size (Splenium: 12729 mm^3^, Body: 13711 mm^3^, Genu: 8851 mm^3^, PLIC R: 3754 mm^3^, PLIC L: 3752 mm^3^, UF R: 380 mm^3^, UF L: 376 mm^3^, CR L: 18077 mm^3^, CR R: 18074 mm^3^, CST R: 1362 mm^3^, CST L: 1370 mm^3^) for all control and mTBI subjects. All regions were verified (by a neuroradiologist) for anatomic accuracy for each scan from each subject. Mean FA, RD, AD and MD were then extracted for each ROI for both mTBI and control subjects.

### Cross-sectional data analysis

We first performed cross-sectional comparisons of DTI metrics from each ROI for both time-points between mTBI and control, using the Mann-Whitney U test, to determine if there were any time-points or regions that might differentiate the groups. We anticipated either no significant differences or differences that were significantly diluted due to noise generated by between-subject variability and injury heterogeneity. Specifically, between-subject variability introduces noise because the normal distribution of DTI metrics, including FA, for a given region is much larger than the magnitude of change attributed to TBI that has been observed in prior human and animal studies [8, 14], and is likely more problematic in mTBI. Further, injury heterogeneity due to different mechanisms, directionality and magnitude of concussive forces likely leads to different white matter tract injuries [15], making it unlikely that changes within a specific ROI would be significant. We do acknowledge that specific regions appear more susceptible to injury and are likely affected by multiple types of injuries, but differences via cross-sectional analysis would regardless be subjected to the noise of injury heterogeneity, albeit to a lesser extent. To control for injury heterogeneity in the cross-sectional analysis, we employed a method reported by MacDonald et al. [8] where we used control subjects to create means and standard deviations for each ROI, which were used to identify the number of abnormal regions in mTBI subjects. We looked for significant differences across all 11 regions for abnormalities rather than each region individually, thus avoiding noise created by regional injury heterogeneity. Abnormal regions were defined as having a DTI metric (FA, MD, RD or AD) >2 standard deviations above or below the mean of controls. When calculating the number of subjects who would be expected by chance to have DTI metrics >2 standard deviations above or below the mean, based on a binomial distribution with n = 11 regions and assuming regions are independent, the difference between groups reaches statistical significance (p<0.05). When we compared the number of mTBI subjects with more than one abnormal region to the number expected by chance based on a binomial distribution, for n = 20 subjects, the difference between groups was not statistically significant (p = 0.0867).

### Longitudinal data analysis

Next, we employed the use of longitudinal data so that within-subjects comparisons could be performed in order to assess changes in DTI metrics across time. By using the changes in DTI metrics to then compare controls and mTBI subjects, we avoided the noise introduced by between-subject variability. When evaluating the longitudinal data, we analyzed the absolute change in the DTI metrics so that both increases and decreases were included, as opposed to just looking unidirectionally or analyzing each separately. We analyzed bidirectional changes because there is no consensus on how DTI metrics change following mTBI, especially longitudinally during the acute post-injury time period, but there is however consensus that these changes likely indicate injury associated with mTBI [15, 16]. There are currently human and animal studies independently showing increases [4, 7] [24] [25] and decreases [26] [8] [27] [28] [29] in FA and other DTI metrics following mTBI, as well as a few showing both increases and decreases [30] [31] [15]. Additionally, there are no longitudinal studies of human mTBI patients, which acquire multiple images, within the first week of injury to suggest how DTI metrics may change in the acute to sub-acute period. Animal studies with longitudinal data acquired during the acute/subacute period following TBI have shown both increases [25] and decreases [27] in FA among white matter tracts following the initial FA changes immediately following injury. While more human [15] and animal studies have found that FA tends to decrease early following injury, they neither account for nor explain why others find contradictory results. Even four of the most recent animal studies using DTI to assess mTBI in the acute period found contradictory changes in FA and other DTI metrics in various white matter regions [24, 27, 28, 30]. Therefore, because both human and animal studies report increases and decreases in FA and other DTI metrics following TBI, we felt that it was important not to limit our study and risk false negative results that could occur with only a unidirectional analysis.

Since current studies suggest increases and decreases occur following TBI, we performed another analysis considering any change in DTI metrics (either increase or decrease) as abnormal. To do this, longitudinal changes in DTI metrics between mTBI and control groups were first calculated across each individual ROI, using the Mann-Whitney U test. Using this method, comparisons between subject variability, but not injury heterogeneity could be accounted for. We predicted that results might reach significance if a specific region was commonly injured or sensitive to injury, or may not reach significance at all if no particular region was injured with high enough frequency. To avoid noise created by injury heterogeneity within the longitudinal data, we again employed a similar approach to MacDonald et al., as we had with the cross-sectional data, by identifying the number of abnormal regions among mTBI subjects. Similarly, abnormal regions were defined as having a DTI metric (FA, MD, RD or AD) >2 standard deviations above or below the mean of controls. Statistical significance was determined by first calculating the number of subjects that would be expected by chance to have DTI metrics >2 standard deviations above or below the mean, based on a binomial distribution of n = 11 regions of interest, assuming regions are independent. Then the number of mTBI subjects with more than one abnormal region were compared to the number expected by chance based on a binomial distribution with n = 20 subjects. Next, we performed the Wilcoxon rank test to compare mean changes in DTI metrics across all 11 ROIs between mTBIs and controls. This allowed for direct comparison between mTBI and controls, while avoiding the noise created by between-subject variability and injury heterogeneity.

### Data reproducibility

To determine the reproducibility of FA measures, both between-subject and within-subject coefficients of variation (CVs) were calculated for the control subjects. The processing stream employs single step resampling of 2mm resolution subject data to 1mm resolution MNI space. Such resampling of data is unlikely to introduce substantial additional smoothness. However, to verify that resampling did not introduce such smoothing, coefficients of variation were calculated for controls a second time by transforming the mask images into the individual space for comparison without resampling of the diffusion data.

### Software

All statistical calculations were performed in SPSS (PASW Statistics 18, release 18.0.2). Matlab was used to analyze the results of genetic programming.

### Genetic programming

Symbolic regression was performed using the genetic programming (GP) package Eureqa [32] to see if we could predict recovery in mTBI patients. This powerful method implicitly combines feature selection, model identification, and parameter estimation, and has been successfully applied in various application domains [33], including identification of nonlinear relationships in BOLD time-series fMRI data between ROIs in the human brain [34]. GP is a population-based algorithm in which sets of candidate solutions (symbolic expressions) are allowed to evolve based on the principles of Darwinian evolution (reproduction with heritable variation, alternating with fitness-based selection). Eureqa is a bi-objective GP that seeks to simultaneously minimize model prediction error and model complexity, and each run returns a set of solutions that are non-dominated with respect to these two objectives. The user can then select solutions that appear to appropriately balance prediction accuracy and parsimony (to minimize the risk of over-fitting). GP is a “white box” optimization algorithm, in that the resultant predictive expressions may provide domain-specific insights. For this study, we sought to evolve functions capable of predicting the sum of post-concussive symptoms at time 2 (S_2_). We specified mean absolute prediction error as the primary objective, and model complexity was defined as the sum of the number of operators, constants, and variables (input features) in each evolved expression. Specifically, we allowed the GP to select and combine: 1) arithmetic operators from the set [18]; 2) co-evolved numerical coefficients; and 3) variables from sets of 1 to 12 possible input features, depending on the particular experiment. Following Eureqa’s recommendations for small data sets, we performed each symbolic regression on all *n* = 36 data points. We considered a total of 34 possible input features: the sum of post-concussive symptoms at time 1 (S_1_) and 3 types of FA data for each of the 11 ROIs. Nine distinct types of experiments were performed using Eureqa (Version 1.24.0), each using a set of 1–12 of these as input features, as detailed in Table 1. For each of the 9 types of experiments, we performed 5 independent *de novo* runs of Eureqa, each starting from random initial populations, to assess consistency of the evolved non-dominated sets of solutions. Each run of Eureqa was manually terminated after Eureqa’s “percent converged” heuristic (based on time since last significant improvement) had reached 100%, which required on the order of 10^9^ function evaluations per run. Running on an 8-core Intel i7-3770 CPU @ 3.40GHz desktop computer, this was usually achieved within 3–5 minutes for the experiments in which the lowest error solutions were achieved. However, in some experiments where there was little or no useful signal in the input feature set, this could take significantly longer (nearly all of the experiments terminated within 30 minutes or less, however one run of experiment 1, which allowed only FAs at time period 1 as input features, required over 2 hours to converge).

**Table 1:** The 9 types of experiments performed with Eureqa were characterized by subsets of the 34 possible input features considered, as outlined here.

Expt #	Experiment Label	Set of Allowable Input Features Presented to Eureqa	# of Input Features
1	FA_1_	FA for all 11 ROI at time1	11
2	ΔFA	FA_1_ –FA_2_ (longitudinal changes) for all 11 ROI	11
3	ΔFA (permuted)	FA_1_ –FA_2_ for all 11 ROI, with subjects’ FA randomly permuted relative to S_1_ and S_2_	11
4	|ΔFA |	|FA_1_ –FA_2_| (absolute value of longitudinal changes) for all 11 ROI	11
5	S_1_	Sum of concussive symptoms at time 1	1
6	S_1_, FA_1_	S_1_ in addition to FA for all 11 ROI at time 1	12
7	S_1_, ΔFA	S_1_ in addition to FA_1_ –FA_2_ for all 11 ROI	12
8	S_1_, ΔFA (permuted)	S_1_ in addition to FA_1_ –FA_2_ for all 11 ROI, with subjects’ FA randomly permuted relative to S_1_ and S_2_	12
9	S_1_, |ΔFA |	S_1_ in addition to |FA_1_ –FA_2_| for all 11 ROI	12

## Results

### Demographics, neurocognitive outcomes and symptoms scores

Subjects included 20 mild TBI patients: 11 male and 9 female, age range 18–57 years, mean age 30.6 years; 16 controls (9 trauma and 7 healthy): 7 male and 9 female, age range 20–57 years, mean age 28.1 years (Table 2). There were no demographic parameters that differed significantly between mTBI and controls. mTBI and extremity trauma control subjects were enrolled in the study and imaged within 72 hours of injury, with a mean time of 46.5±22 hours for mTBI and 57.6±12 hours for the controls. All subjects from the mTBI and control groups returned for follow-up imaging one week later, at a mean of 6.7±1.1 days for mTBI and 6.9±1.7 days for all controls. All mTBI subjects were symptomatic at the time of first imaging. There were no differences in head motion between mTBI and controls groups. mTBI subjects reported significantly more symptoms at time-points 1 and 2 compared to controls. We observed a wide variation in reaction time, working memory and other neurocognitive testing results among both the injured groups and controls, but differences in functional measurements between groups were not significant (Table 3).

**Table 2:** Demographics. The control group included 9 extremity injured patients and 7 healthy controls. *Injury to time of MRI 1 and 2 is only applicable to the 9 extremity injured patients.

	mTBI (n = 20)	Control (n = 16)	Difference (95% CI)
Gender (n (%) male)	11 (55%)	7 (44%)	
Handedness (n (%) right handed)	17 (85%)	13 (81%)	
Age (years)	30.6	28.1	2.5 (-5.5,10.5)
Education (years)	14.7	15.7	-0.8 (-2.3,0.7)
Injury to MRI 1 (days)	1.9	2.4	-0.5 (-1.2,0.2)
Injury to MRI 2 (days)	8.6	9.3	-0.7(-1.9,0.4)
MRI 1 to MRI 2 (days)	6.7	6.9	-0.2(-1.3,0.9)

**Table 3:** Neurocognitive and symptom outcomes for the mTBI subjects and trauma controls. Outcomes were measured in those controls subjects with extremity injuries only. Values given as mean ± standard deviation.

*Total Symptom Score*	mTBI (n = 20)	Controls* (n = 9)	Difference (95% CI)
# Symptoms (Time 1)	20.9 ± 18.4	3.3 ± 5.7	17.6 (8.2,27.0)
# Symptoms (Time 2)	10.3 ± 15.1	2.1 ± 3.4	8.2 (0.8,15.6)
***Neurocognitive Testing Score***			
Verbal Memory (Time 1)	84.6 ± 11.7	80.4 ± 12.7	4.3 (-6.1,14.6)
Visual Memory (Time 1)	69.7 ± 18.7	67.3 ± 15.0	2.5 (-11.9,17.0)
Visual Motor Speed (Time 1)	41.4 ±10.2	36.6 ± 8.2	4.8 (-3.1,12.7)
Reaction Time (Time 1)	0.63 ± 0.16	0.58 ± 0.11	0.05 (-0.06,0.17)
Impulse Control (Time 1)	3.9 ± 3.2	14.9 ± 28.4	-11.0 (-34.8,12.8)
Cognitive Efficiency Index (Time 1)	0.27 ± 0.21	0.21 ± 0.20	0.06 (-0.12,0.25)
Verbal Memory (Time 2)	91.5 ± 8.2	81.0 ± 16.5	10.5 (-2.5,23.4)
Visual Memory (Time 2)	68.7 ± 14.2	70.9 ± 16.5	-2.2 (-15.9,11.5)
Visual Motor Speed (Time 2)	41.8 ± 10.4	39.2 ± 8.3	2.6 (-5.0,10.2)
Reaction Time (Time 2)	0.59 ± 0.11	0.58 ± 0.15	0.01 (-0.11,0.14)
Impulse Control (Time 2)	5.2 ± 4.6	15.2 ± 33.0	-10.1 (-35.5,15.3)
Cognitive Efficiency Index (Time 2)	0.36 ±0.20	0.34 ± 0.21	0.02 (-0.16,0.20)

### Reproducibility of diffusion measures

DTI measurements of white matter in healthy individuals across time were remarkably stable. The ROI for the Left Uncinate Fasciculus did not anatomically fit one control subject, so that region was therefore excluded from analysis for that individual.

FA measurements across the 11 regions of interest showed that the between-subject variation (CV range 2.3% to 7.3%) was far greater than within-subject variation (CV range 0.4% to 4%). Through the use of longitudinal data, a 70–97% reduction in variance can be achieved (Table 4). CVs calculated in the native space of the control subjects showed negligible differences compared to the CVs calculated from the resampled control data used for analysis (S6 Table). Large white mater regions with higher FA values were also found to have lower levels of within-subject variation. Within our control population, we found a high degree of reproducibility across 1 week (for individual subjects at two time points R^2^ = 0.971).

**Table 4:** Within- and between-subject variation in FA seen in control subjects across 1 week. For control subjects, the standard deviation of FA values for each ROI at time point 1 and 2, along with the standard deviation of change within subjects across the two time points is shown. The final column shows the percent reduction in variation achieved through the use of longitudinal opposed cross-sectional data for each ROI.

Region	Mean FA	Between-Subject				Within-Subject		Proportion of variance due to between-subject variability (%)
		Scan 1		Scan 2				
		Std Dev	CV (%)	Std Dev	CV (%)	Std Dev	CV (%)	
Splenium CC	0.69	0.016	2.3	0.017	2.5	0.003	0.4	96.8
Body CC	0.56	0.026	4.6	0.028	5.0	0.006	1.0	95.5
Genu CC	0.52	0.015	2.9	0.016	3.1	0.005	1.0	89.7
PLIC (right)	0.57	0.017	3.0	0.016	2.8	0.008	1.4	79.4
PLIC (left)	0.58	0.016	2.8	0.016	2.8	0.006	1.0	88.0
UF (right)	0.43	0.027	6.3	0.025	5.8	0.011	2.6	82.3
UF (left)	0.40	0.027	6.8	0.029	7.3	0.016	4.0	70.2
CR (right)	0.42	0.013	3.1	0.015	3.6	0.004	1.0	92.6
CR (left)	0.42	0.017	4.0	0.018	4.2	0.004	1.0	93.6
CST (right)	0.52	0.033	6.3	0.034	6.5	0.011	2.1	88.0
CST (left)	0.52	0.032	6.3	0.033	6.3	0.012	2.3	87.0

Corpus callosum (CC), posterior limbs of the internal capsule (PLIC), uncinate fasciculus (UF), corona radiata (CR) and corticospinal tract (CST).

### Cross-sectional analysis

We did a cross-sectional analysis looking at the two time-points individually. We compared DTI measures in the 11 ROIs between mTBI to control, and we also compared the total number of ROIs that were abnormal between mTBI and control.

#### mTBI vs control region of interest analysis

**Fig 1:**
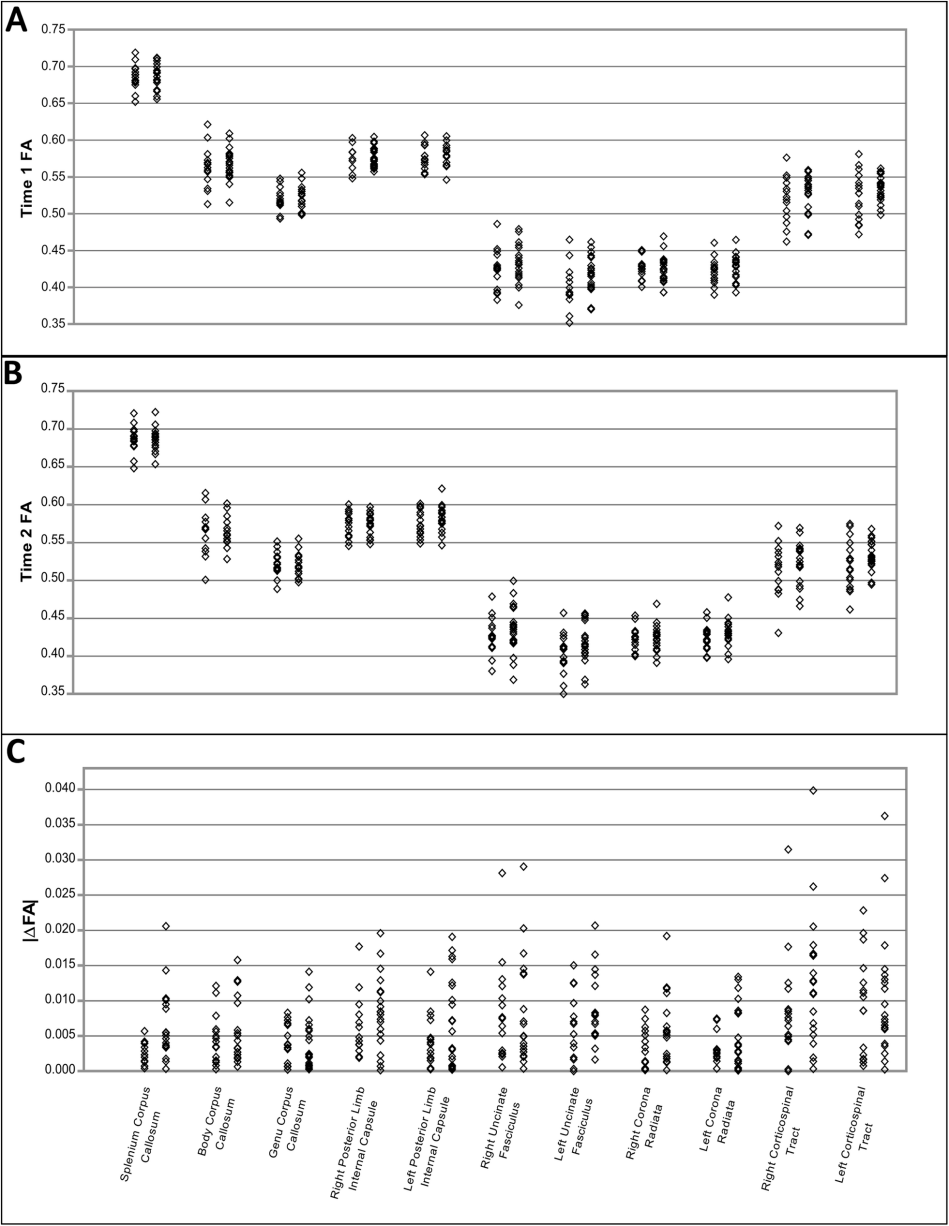
Longitudinal analysis of DTI metrics provides better discrimination of mTBI from controls than imaging at a single time-point. For each ROI the controls and mTBIs are separated with the controls on the left and the mTBIs on the right. (A) FA values for time point 1. (B) FA values for time point 2. (C) Absolute change in FA values.

Of the 11 ROIs, no significant (p>0.05) differences between mTBI and control subjects were found at either time-point 1 or 2 for any of the quantifiable DTI measures (FA, MD, RD, AD) (S1–S4 Tables) (Fig 1A and 1B),

#### mTBI vs control analysis of number of abnormal regions analysis

The number of abnormal ROIs for only one of the DTI measurements, AD, was significantly increased compared to controls. This was only true for time-point 1 (p = 0.025), and not time-point 2 (S4A and S4B Fig). No other DTI metrics showed significantly increased numbers of abnormal regions at either time-point (S1A, S1B, S2A, S2B, S3A, S3B, S4A and S4B Figs).

### Longitudinal analysis

We then looked at the change in DTI measures between the two time-points.

#### mTBI vs control region of interest analysis

When comparing the absolute change between time 1 and time 2, we found the largest difference in the splenium of the CC in mTBI subjects compared to controls (p<0.05) (Table 5) (Fig 1C). All ten remaining ROIs did not differ significantly compared to controls. When collectively comparing all eleven ROIs, the mean FA change was significantly greater in the mTBI subjects than in the control group (p<0.05, Wilcoxon signed rank test). Further, 16 out of the 20 mTBI subjects were found to have at least one region, and as many as 7 regions, where the change in FA exceeded the changes seen across all controls within specific regions. The quantitative diffusion measurements of MD, RD, and AD comparing absolute changes across time-points are provided in supplemental tables (S2–S4 Tables).

**Table 5:** Distribution of absolute changes in FA (×10^−3^) between mTBI and control subjects over a one-week period. Values are given as mean ± standard deviation.

Region of Interest	mTBI	Control	Difference (95% CI)	P-value (uncorrected)
Splenium CC	6.1 ± 4.9	2.6 ± 1.5	3.57 (1.16,5.98)	0.009
Body CC	5.9 ± 4.8	4.4 ± 3.5	1.46 (-1.35,4.26)	0.464
Genu CC	4.2 ± 4.1	4.2 ± 2.7	0.06 (-2.25,2.36)	0.588
PLIC (right)	8.2 ± 5.2	6.2 ± 4.1	2.00 (-1.16,5.10)	0.161
PLIC (left)	7.3 ± 6.4	4.3 ± 3.6	2.92 (-0.53,6.37)	0.340
UF (right)	8.1 ± 7.6	8.0 ± 7.0	0.08 (-4.87,5.03)	0.849
UF (left)	8.7 ± 5.0	5.9 ± 4.9	-2.78 (-6.20,0.64)	0.099
CR (right)	5.6 ± 4.8	3.3 ± 2.7	2.32 (-0.28,4.92)	0.181
CR (left)	5.5 ± 4.8	3.7 ± 2.5	1.81 (-0.70,4.35)	0.426
CST (right)	12.5 ± 9.4	8.7 ± 7.5	3.79 (-1.94,9.51)	0.152
CST (left)	10.5 ± 8.8	9.4 ± 7.1	1.10 (-4.30,6.50)	0.849

Corpus callosum (CC), posterior limbs of the internal capsule (PLIC), uncinate fasciculus (UF), corona radiata (CR) and corticospinal tract (CST).

#### mTBI abnormal regions analysis

**Fig 2:**
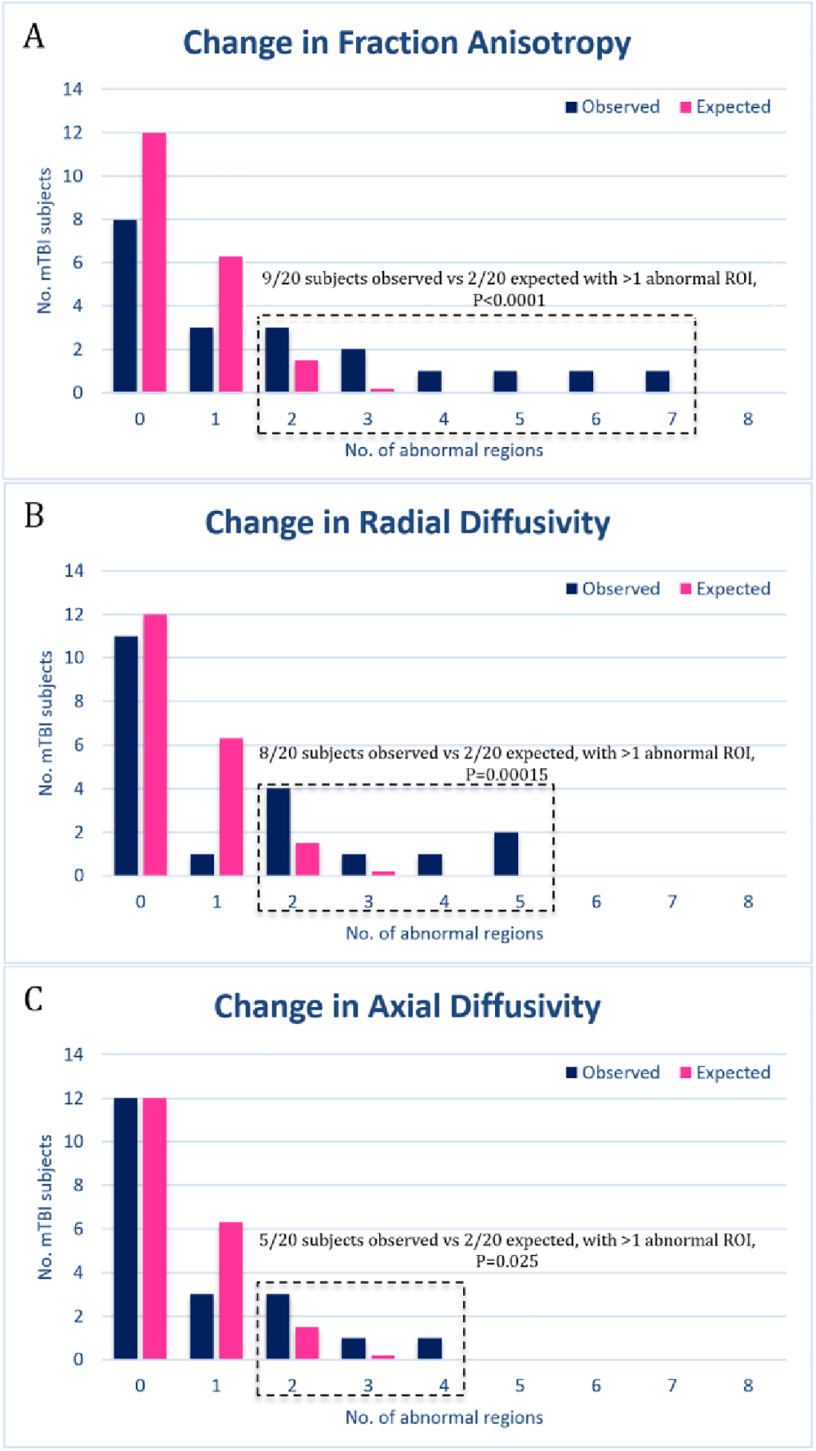
Number of abnormal regions of interest in mild traumatic brain injury (mTBI) subjects. Abnormal regions are defined as having DTI metrics more than 2 standard deviations above or below the mean for the control group. Blue bars indicate the number of mTBI subjects with a given number of abnormal regions. Pink bars indicate the number of subjects that would be expected by chance, based on a binomial distribution with n = 11 regions, p = 0.0455. Regions are assumed to be independent. Dashed boxes indicates metrics in which the number of mTBI subjects with more than one abnormal region is significantly different to that expected by chance (binomial distribution, n = 20 subjects, p = 0.0867) [8]. (A) Number of abnormal changes in fractional anisotropy in mTBI subjects. (B) Number of abnormal changes in radial diffusivity. (C) Number of abnormal changes in axial diffusivity.

FA, RD and AD all showed significantly increased abnormal changes in ROIs across time-points. When quantifying FA, 9/20 subjects were found to have more than 1 region with abnormal changes (p<0.05). RD found 8/20 subjects with more than 1 region with abnormal change (p<0.05), and AD found 5/20 subjects with more than 1 region with abnormal change (p<0.05). MD did not show a significantly increased number of abnormal changes among the 11 regions (Fig 2).

### Genetic programming

Finally, we performed a post-hoc exploration of our data to identify correlations between DTI measures and clinical outcome variables that may be tested in future studies. We computed the Pearson correlation coefficient (r) between each of the 34 possible input features provided to Eureqa and the outcome variable S_2_. Of these, only 9 had statistically significant correlations (p ≤ 0.05), as shown in Table 6 Not surprisingly, the strongest of these correlations was between S_1_ and S_2_. More interesting were the significant correlations we found between S_2_ and (i) the FA of region UF (left) at time period 1, (ii) longitudinal changes in the FA of CR-L_All, and (iii) absolute values of longitudinal changes in FA in 6 other ROIs.

**Table 6:** Significant pearson correlation coefficients were found between 9 of the 34 possible input features provided to Eureqa and the outcome variable S_2_. The notation ΔFA means FA_1_ –FA_2_.

feature	r	R^2^	p-value
|Δsplenium|	0.488	0.238	0.0025
|Δbody|	0.332	0.110	0.0477
|Δgenu|	0.348	0.121	0.0375
|ΔPLIC-L|	0.449	0.201	0.0060
|ΔCR-R_All|	0.470	0.221	0.0038
|ΔCST-R|	0.438	0.192	0.0076
ΔCR-L_All	0.342	0.117	0.0409
UF_L_1_	0.329	0.108	0.0498
S_1_	0.658	0.433	1.318e-5

**Fig 3:**
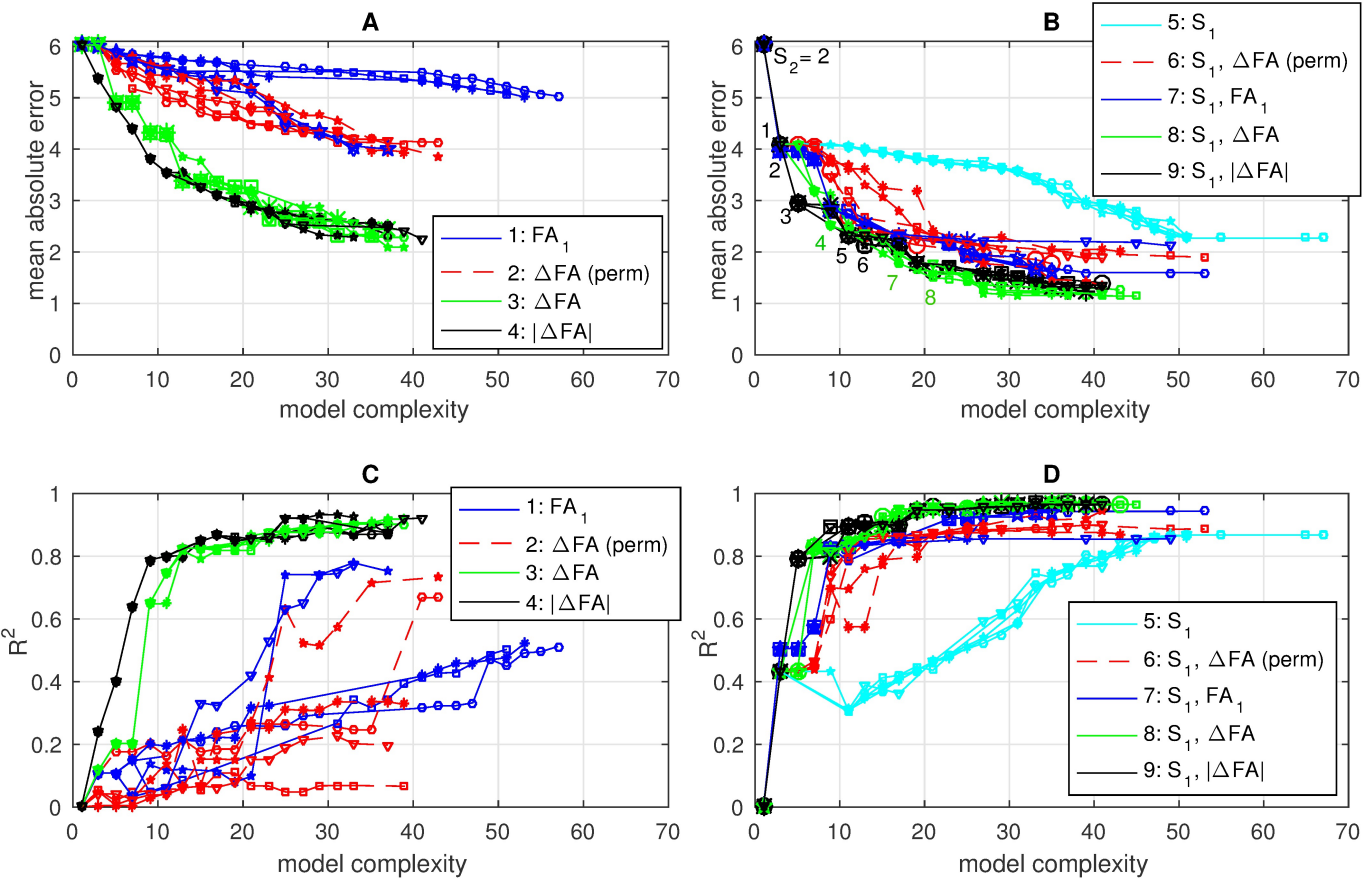
Non-dominated fronts of error vs. complexity resulting for 5 independent Eureqa runs for each of experiments 1–4 (A) and 5–9 (B), with corresponding adjusted R^2^ values relating the evolved expressions to S_2_ shown in panels (C) and (D), respectively. The complexity 1 expression was always the constant expression S_2_ = 2. The expressions corresponding to the 8 numbered points in (C) are detailed in Table 7.

**Fig 4:**
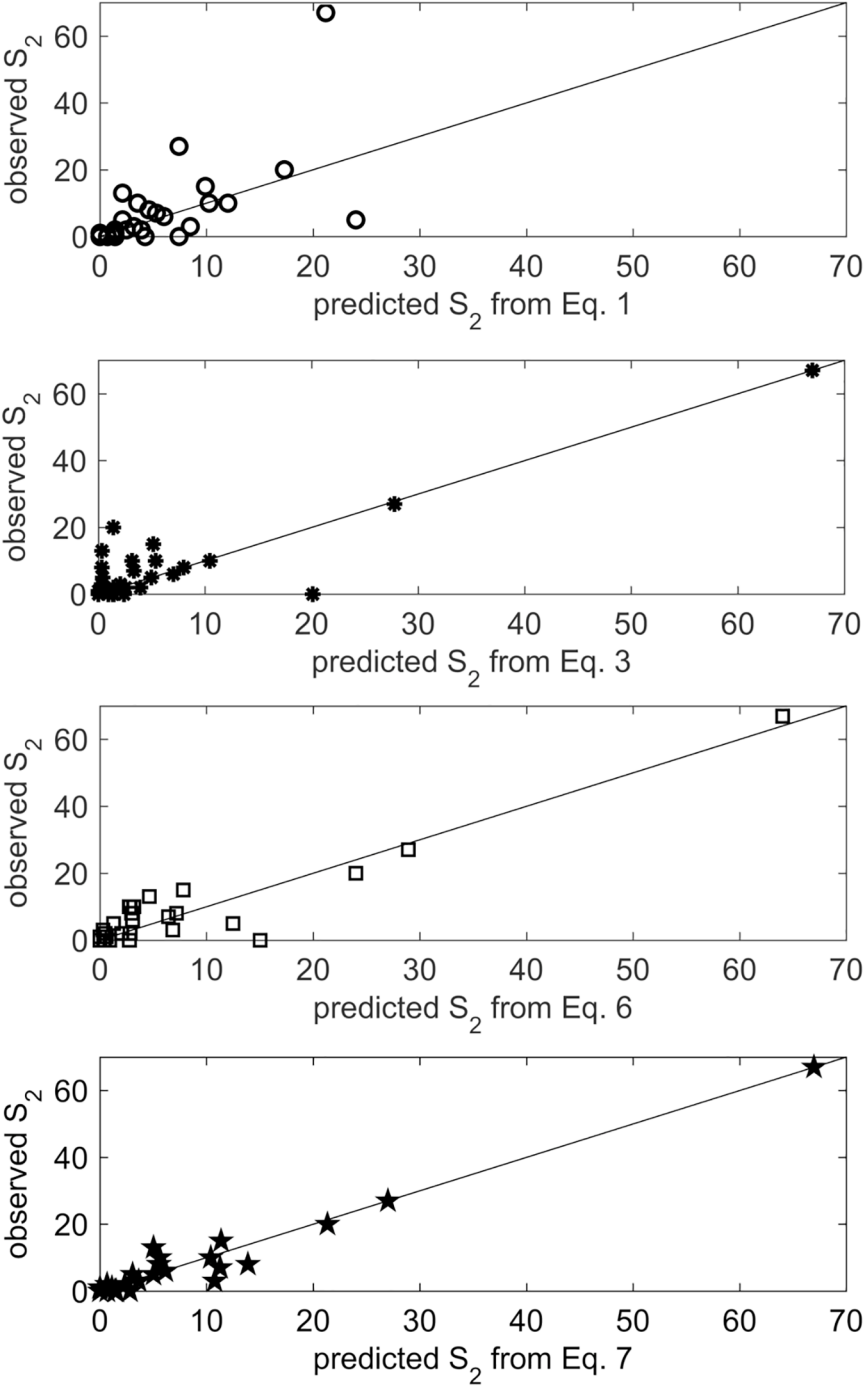
Observed vs. predicted sum of post-concussive symptoms at time period 3, shown for 4 of the evolved expressions shown in Table 6. The top one is simply the linear relationship with S_1_, the middle two were evolved from experiment 9 (using S_1_ and |ΔFA| as input features), and the bottom one was evolved from experiment 8 (using S_1_ and ΔFA as input features).

However, Eureqa was able to evolve many expressions with interaction terms and/or higher-order terms that exhibited much higher correlations with S_2_. Of the 561 possible pairs of the 34 features, 98 of them had significant cross-correlations (p < 0.05); we hypothesized that taking products of some of these features may have amplified the signal and provided complementary information. Fig 3 shows the resulting non-dominated fronts of error vs. complexity resulting from all 9 experiments, and the corresponding adjusted R^2^ values. In all experiments, the simplest (complexity 1) expression on the left was always the constant expression S_2_ = 2, which is simply the average S_2_. At the other extreme, the most complex and accurate solutions at the right are the result of extreme over-fitting. Details on the 8 most promising expressions along the non-dominated front shown in Fig 3B are given in Table 7, and for 4 of these expressions we plot predicted vs. observed (Fig 4).

**Table 7:** Expressions corresponding to the 8 numbered points on graph C.

Eq. #	Evolved expression	Mean abs error	Complexity	Adjusted R^2^	Found in Experiments	Frequency Found
1	S_2_ = 0.353*S_1_	4.103	3	0.43	2–6,8–9	20/20 runs
2	S_2_ = S_1_*(UF-L_1_)	3.982	3	0.51	7	5/5 runs
3	S_2_ = 93.73* S_1_*|Δgenu|	2.936	5	0.79	9	5/5 runs
4	S_2_ = 0.346* S_1_ + Δbody* S_1_^2^	2.513	9	0.82	8	4/5 runs
5	S_2_ = 39.25* S_1_*(|CR-L_All|) + |Δsplenium|* S_1_^2^	2.312	11	0.90	9	5/5 runs
6	S_2_ = 52.05* S_1_*|Δsplenium| + 0.7577*(|CR-L_All|)* S_1_^2^	2.146	13	0.88	9	3/5 runs
7	S_2_ = 0.282* S_1_ + 36.90* S_1_*Δbody + 3489.1* S_1_*Δsplenium^2^	1.776	17	0.92	8	4/5 runs
8	S_2_ = 0.356* S_1_ + 3.051e8*(ΔPLIC-R)* Δsplenium^3^ + 0.868*Δbody* S_1_^2^	1.491	21	0.96	8	1/5 runs

We first addressed the results of experiments 1–4 (Fig 3A and 3C) which used only FA data for input features. In experiment 1, in which the only input feature was FA at time period 1 (blue lines), the very gradual decline in error with increasing complexity in the results indicate that there is little or no useful signal in this data alone that is predictive of S_2_. On the other hand, regressing on ΔFA values (green lines) gave a much sharper drop in error than regressing on the same ΔFA values but with rows permuted (red lines), indicating that there is some useful signal included in the ΔFA values that is predictive of S_2_. The models resulting from regressing on |ΔFA| values (black lines) exhibited even lower errors and higher R^2^ values than when regressing on ΔFA, and there was greater consistency between the 5 independent model runs using |ΔFA|, with an apparent “knee” (change in slope) in both error and R^2^ at model complexity 9.

Not surprisingly, the errors in models from experiments 5–9, which were all evolved using S_1_ as an input feature (Fig 3B and 3D), were much lower than models of the same complexity that were evolved without using S_1_ (Fig 3A), since S_1_ had the largest main effect (Table 6). When S_1_ is the only input feature (Fig 3B, cyan lines), there is a sharp drop in error between the constant model and the linear model (Eq 1 in Table 7), but adding in higher order terms only reduces the error gradually, due to over-fitting. Similarly, including permuted ΔFA values (experiment 6, red lines), where we had randomized the association between features and outcomes, causes gradual decreases in error due to over-fitting. However, including FA_1_ (experiment 7, blue lines) appears to provide a small amount of additional signal. In fact, the equation numbered 2 actually dominates the linear model of the equation numbered 1 (Table 7). The best models were achieved with using S_1_ in conjunction with either ΔFA (experiment 8, green lines) or |ΔFA| (experiment 9, black lines), as shown with the non-dominated equations numbered 3–8 (Table 7). Interaction terms in these expressions appear to be boosting the useful signal in these data. Again, the results were more consistent between runs when using |ΔFA| and, for the more parsimonious models (of complexity ≤ 13), the R^2^ values were also higher when using |ΔFA|, indicating that there is more useful signal in these data.

## Discussion

This neuroimaging study of emergency department patients with acute head injury, with imaging immediately after injury and one week later, has several important implications. First, we show that longitudinal analysis of changes in FA in each ROI, using each individual subject as their own control, discriminated between mTBI subjects compared to controls, identifying abnormalities which would have not been detected using standard cross-sectional and whole brain analysis approaches. This corroborates prior findings of diffusion changes occurring across time following TBI, which have been described in human subject studies that have used longitudinal imaging, but over a longer time period [11, 26, 35]. Our study is significant because it is the first to have employed multiple timepoint imaging for both mTBI and control subjects within the first week after injury. We validate previous studies showing that ROI-based analysis of FA measurements provides the highest sensitivtiy for detection of axonal injury in mTBI. We also demonstrated that inclusion of all *a priori* ROIs in an analytic model as opposed to measuring individual ROIs improves detection of white matter changes by overcoming issues of injury heterogeneity. Finally, we use a genetic programming approach to provide support for a model associating longitudinal changes in FA with the severity of post-concussive symptoms 7–10 days after mTBI In this model, the direction (increase or decrease in FA value) was not as predictive as the absolute change in FA. In sum, identification of white matter ROIs with changes in FA signal, over the week after trauma, may provide critical insight into understanding the clinical effects of concussive head injury.

There is no established consensus for optimal, quantitative DTI analysis to identify mTBI. Region of interest (ROI) methods are generally used, but this technique may artifactually minimize group differences when ROIs are placed within maximal FA regions on post-processed FA maps and underestimate FA values if ROIs are placed adjacent to low-FA structures where partial volume averaging artifacts occur such as gray-white junctions or the pericallosal white matter. Voxel-based methods such as tract-based spatial statistics that normalize DTI data to a common space to account for differences in individual brains (i.e., size and shape) are subject to error from partial volume effects, particularly as slice thickness is increased. “Pothole” techniques [11, 36–40], in which areas of unusually high or low FA are identified throughout the brain, and the number and/or volume of such regions provides a single metric of injury, has been shown to result in large numbers of false-positives due to the issue of multiple comparisons, and recent research has questioned aspects of its methodology used in many TBI studies [41]. In our study, we compared FA values to regions of the brain most commonly associated with mTBI [16, 42] to provide a proxy measure of injury severity without introducing the issues of multiple comparisons to the extent seen in voxel-based analyses that may lead to false positive errors.

We demonstrate that in our cohort, counting the total number of abnormal ROIs in individual patients at either time point was not sensitive to detection of mTBI. There were a significant number of abnormal regions in only one DTI metric, AD, among mTBI subjects, but only at time-point 1. Otherwise no significant differences were found at either individual time-points or for any individual ROI whether compared cross-sectionally or longitudinally. We observed some ROIs, such as the splenium of the CC, trended towards showing a significant difference in the comparison between groups, but these differences were not statistically significant when corrected for multiple comparisons. These findings demonstrate the impact that inter-subject variability and injury heterogeneity have on diluting subtle white matter changes seen following mTBI. We acknowledge that there are a number of studies demonstrating significant findings using cross-sectional ROI analyses, but many of these results may be explained by similarity of injury mechanism among the study population and/or increased severity of injury [8, 15]. Further, based on our findings, we suggest results of prior studies may be missing significant white matter changes due to analysis methodology that is not sensitive enough to subtle changes in white matter seen across time. If these subtle changes play significant roles in the initial and long-term symptoms seen in mTBI, as we have suggested, then this may explain why DTI metrics have thus far been poorly correlated with mTBI symptoms and prognoses [15, 16].

Studies using whole-brain analyses are also susceptible to the effects of inter-subject variability, but to a larger extent avoid the issue of injury heterogeneity. This allows for improved ability to detect white matter changes and may explain why the analysis method has been popular in recent years. Our results however suggest that these studies likely still fail to capture the true degree and extent of white matter changes, thereby demonstrating an incomplete picture of the white matter disruption caused by mTBI. Our results support and extrapolate on the earlier discovery by Inglese et al. that abnormalities in FA and MD after mTBI are too subtle to be detected by whole brain analysis [43].

It also is worth considering whether FA is the most appropriate diffusion metric for assessing mTBI. Inglese et al. [43], performed a cross-sectional study of whole brain and ROI analysis in subjects after mTBI. Similar to our results, they found diffusion changes in FA within commonly injured white matter regions such as the CC and internal capsule, which were not detected by whole brain analysis. They concluded that early time-point imaging may have utility as a prognostic measure, consistent with our genetic programming analysis. Our findings differ with regards to the utility of MD; AD and RD had more diagnostic utility, but none of these measures provided much additional discriminatory capacity to distinguish mTBI patients from controls. Clinical MR DTI sequences model diffusion as a single three-dimensional ellipsoid, but newer methodologies, such as CHARMED [44] (composite and restricted model of diffusion), which account for different compartments, may be more realistic and better differentiate edema from axonal disruption. Most tensor models do not account for the presence of multiple fiber populations within a single voxel, but instead average them together, resulting in an apparent decrease in FA. In addition, heterogeneity of the injuries and forces that lead to mTBI as well as the spatial heterogeneity of damage within the brain parenchyma [13] ultimately limit the ability of DTI in the setting of acute mTBI regardless of the diffusion metric used. FA was the most sensitive measure in our study for capturing white matter changes in mTBI subjects. These findings are consistent with most current animal and human studies and demonstrate the importance of employing multiple DTI metrics during analysis for future studies [15, 16, 30]. Our study corroborates the importance of DTI with ROI analysis of FA to identify axonal injury in mTBI subjects, and further suggests that analysis of longitudinal imaging across time may detect more subtle white matter injury than single time point analysis.

Furthermore, on closer examination of the DTI changes demonstrated via longitudinal analysis, that most subjects only have a few regions with significant changes in white matter; rather than a more diffuse pattern across all ROIs. We believe that this further demonstrates the focal and heterogeneous nature of white matter injury after in mTBI, which is likely secondary to variables such as injury mechanism, location, directionality and magnitude of impact. Our results show that, for every ROI analyzed, the subject with the greatest degree of change in FA for that ROI was always from the mTBI group. When looking across individuals, 80% of the mTBI subjects exhibited one or more regions with a change in greater magnitude than all changes seen in controls for that region. These findings provide convincing evidence that these changes are markers of white matter disruption. The reason 80% and not 100% of mTBI subjects had a larger change than controls may be due a variety of factors including injury severity, imaging timepoints in conjunction with the time frame of specific patient’s recovery or that the location of injury was within an uncommon white matter tract. We also noted that certain regions such as the splenium of the CC had an increased frequency of white matter changes among mTBIs, likely representing an anatomical susceptibility as has already been suggested by prior research [16]. Moreover, the regions most commonly associated with FA changes in our study were consistent with those previously published [16].

In addition, we observed both increases and decreases in DTI signals among mTBI subjects following injury, which matches findings in both animal and human literature [15, 16, 30]. The variability in directionality of DTI metrics likely involves co-occurring processes of injury and recovery [15, 30]. While there currently is no consensus on the true mechanism behind the injury and recovery processes occurring within white matter following mTBI, the most recent animal models have proposed the changes are driven by edema, gliosis, inflammatory cytokines, and various astrocytic processes [24, 27, 28, 30]. All of these likely vary among individuals due to a large number of factors including severity of injury and individual recoverability.

Our results also demonstrate the highly reproducible nature of ROI-based FA measurements in human subjects across short periods of time. Longitudinal imaging in our control population enabled us to determine coefficients of variation (CV) for test-retest reliability. The CVs determined from our control subjects compared well to those published previously [45]. For large, uniform white matter structures such as the splenium, the within-subject CV of 0.4% is remarkably good. Smaller anatomic structures such as the uncinate fasciculus have substantially higher CVs. Because the higher sensitivity of the longitudinal study is likely due to the fact that within-subject variability is much smaller that between-subject variability, technical improvements such as multiband imaging [46] may enable faster, more accurate, and reproducible determination of DTI metrics, but are unlikely to substantially increase sensitivity in cross-sectional studies where between-subject variability is the dominant source of uncertainty. For longitudinal studies, within-subject reproducibility is the limiting factor, and technical improvements may improve sensitivity.

Not surprisingly, we found that the sum of post-concussive symptoms at time 1 (S_1_) by itself had a moderate linear relationship (R^2^ = 0.43) with the sum of post-concussive symptoms at time 2 (S_2_). However, when we evolved non-linear expressions using Genetic Programming (GP) that combined S_1_ with longitudinal changes in FA, we found several expressions with relatively low model complexity (indicating they were not simply over-fitting the data) that were much more strongly predictive of S_2_ (with R^2^ values up to 0.96). Furthermore, the observation that we could not evolve such strongly predictive models when we randomly permuted the longitudinal changes in FA from each patient, relative to the observed values of S_1_ and S_2_, is another indication that there is meaningful signal in the longitudinal changes in FA. Finally, in the more parsimonious evolved models, we observed greater consistency and greater predictive value when using the absolute value of longitudinal changes in FA. Thus, although with only 36 data points, these results from the GP are far from conclusive, they do offer support for the following two conjectures:

1. There is useful signal in longitudinal changes in FA that is associated with the severity of post-concussive symptoms 7–10 days after mTBI; and
2. Using absolute values of longitudinal changes in FA appears to have slightly more useful signal than using signed changes, especially at low model complexities, where over-fitting is less likely.

Limitations of our study include the relatively small sample size and the use of only two time points in the acute and subacute phases, as opposed to an additional third point in the chronic phase. A recent meta-analysis [15] showed that FA increases are generally seen in the acute stage 2–4 days post-injury, changes are inconsistent in the sub-acute stage of 4 days-2 weeks post-injury, and decreases are observed beyond 2 weeks. In our study, we observed greater change in the mTBI group compared to the control group. Directions of change varied in different ROIs. Whether longitudinal imaging is more sensitive than cross-sectional imaging may depend on the magnitude of change in DTI metrics over the time course between scanning. Further work is required to determine optimal imaging time points to maximize the sensitivity of longitudinal studies. Another limitation is the analysis of only 11 white matter regions where injury was most commonly reported. However, because only 11 regions were analyzed, it is likely that mTBI subjects had injury to additional regions, and it is possible that some mTBI subjects had no injury among our 11 chosen regions. It is also important to address the fact that changes in FA among our study population are relatively small in magnitude compared to overall FA values. While we acknowledge this fact, it is important to remember that because we have demonstrated, as have other studies [22], that FA is highly reproducible across time in healthy individuals, that significant changes would still hold clinical significance. Further, subtle abnormalities are rather, to an extent, actually anticipated due to the variable nature of injury within the mTBI population. Furthermore, when examining large white matter tracts, it would be paradoxical to theorize that large-magnitude changes/insults, which commonly present with more severe neurological findings consistent with stroke or severe TBI, would produce the clinically non-specific symptomatology seen in mTBI.

### Practical applications

Significant changes in FA were detected in patients with mTBI within the first week following injury. Although between-subject variation is a substantial obstacle in the study of mTBI using DTI, we demonstrate that longitudinal imaging over the first week after an injury improves the characterization of mTBI by DTI metrics. Our results suggest that sequential imaging of the same individual is superior to cross-sectional imaging for quantitative DTI analysis of mTBI. Our results using genetic programming support this suggestion that longitudinal changes in FA may have clinical utility in predicting severity of post-concussive symptoms a week or more after mTBI, and that the magnitude of change may be more predictive of long-term outcomes compared to the directionality of the change.

## Supporting information

S1 Fig Abnormal regions in fractional anisotropy. (A) Time 1, (B) Time 2 and (C) Change over time.
(TIF)

S2 Fig Abnormal regions in mean diffusivity. (A) Time 1, (B) Time 2 and (C) Change over time.
(TIF)

S3 Fig Abnormal regions in radial Diffusivity. (A) Time 1, (B) Time 2 and (C) Change over time.
(TIF)

S4 Fig Abnormal regions in axial Diffusivity. (A) Time 1, (B) Time 2 and (C) Change over time -4 *for S1–S4 Figs. Number of abnormal regions of interest in mild traumatic brain injury (mTBI) subjects. Abnormal regions are defined as having DTI metrics more than 2 standard deviations above or below the mean for the control group. Blue bars indicate the number of mTBI subjects with a given number of abnormal regions. Red bars indicate the number of subjects that would be expected by chance, based on a binomial distribution with n = 11 regions, p = 0.0455. Regions are assumed to be independent (8). Dashed boxes indicates metrics in which the number of mTBI subjects with more than one abnormal region is significantly different to that expected by chance (binomial distribution, n = 20 subjects, p = 0.0867).
(TIF)

S1 Table Mean FA values for TBI and control subjects at time 1, time 2 and delta. (DOCX)

S2 Table Mean MD values for TBI and control subjects at time 1, time 2 and delta. (DOCX)

S3 Table Mean RD values for TBI and control subjects at time 1, time 2 and delta. (DOCX)

S4 Table Mean AD values for TBI and control subjects at time 1, time 2 and delta. (DOCX)

S5 Table Mechanisms of injury. (DOCX)

S6 Table Reproducibility of FA seen in control subjects without resampling across 1 week. Binary masks defining each region of interest were transformed into the individual subject space using nearest neighbor resampling. This prevented any possible effect of smoothing of the original data, although the mask definitions are likely to be somewhat less accurate. For control subjects, the standard deviation of FA values for each ROI at time point 1 and 2, along with the standard deviation of change within subjects across the two time points is shown.
(DOCX)

S1 Methods Supplemental methods. (DOCX)

S1 Dataset Original DTI metrics data. (XLSX)

## References

[1] Marin JR, Weaver MD, Yealy DM, Mannix RC. Trends in visits for traumatic brain injury to emergency departments in the United States. JAMA: the journal of the American Medical Association. 2014;311(18). doi: 10.1001/jama.2014.3979 24825648 10.1001/jama.2014.3979

[2] Faul MX, L.; Wald, M.M.; Coronado, V.G. Traumatic Brain Injury in the United States: Emergency Department Visits, Hospitalizations and Deaths 2002–2006. Centers for Disease Control and Prevention, National Center for Injury Prevention and Control. 2010;Atlanta (GA).

[3] Arciniegas DB, Anderson CA, Topkoff J, McAllister TW. Mild traumatic brain injury: a neuropsychiatric approach to diagnosis, evaluation, and treatment. Neuropsychiatric disease and treatment. 2005;1(4):311–27. Epub 2008/06/24. ; PubMed Central PMCID: PMC2424119. 18568112 PMC2424119

[4] Sharp DJ, Ham TE. Investigating white matter injury after mild traumatic brain injury. Current opinion in neurology. 2011;24(6):558–63. Epub 2011/10/12. doi: 10.1097/WCO.0b013e32834cd523 . 21986682 10.1097/WCO.0b013e32834cd523

[5] Nickerson JP, Koski CJ, Boyer AC, Burbank HN, Tandan R, Filippi CG. Linear longitudinal decline in fractional anisotropy in patients with amyotrophic lateral sclerosis: preliminary results. Klinische Neuroradiologie. 2009;19(2):129–34. Epub 2009/07/29. doi: 10.1007/s00062-009-8040-1 . 19636503 10.1007/s00062-009-8040-1

[6] Arfanakis K, Haughton VM, Carew JD, Rogers BP, Dempsey RJ, Meyerand ME. Diffusion tensor MR imaging in diffuse axonal injury. AJNR American journal of neuroradiology. 2002;23(5):794–802. Epub 2002/05/15. . 12006280 PMC7974716

[7] Mayer AR, Ling J, Mannell MV, Gasparovic C, Phillips JP, Doezema D, et al A prospective diffusion tensor imaging study in mild traumatic brain injury. Neurology. 2010;74(8):643–50. doi: 10.1212/WNL.0b013e3181d0ccdd ; PubMed Central PMCID: PMC2830922. 20089939 10.1212/WNL.0b013e3181d0ccdd PMC2830922

[8] Mac Donald CL, Johnson AM, Cooper D, Nelson EC, Werner NJ, Shimony JS, et al Detection of blast-related traumatic brain injury in U.S. military personnel. The New England journal of medicine. 2011;364(22):2091–100. Epub 2011/06/03. doi: 10.1056/NEJMoa1008069 ; PubMed Central PMCID: PMC3146351. 21631321 10.1056/NEJMoa1008069 PMC3146351

[9] Aoki Y, Inokuchi R, Gunshin M, Yahagi N, Suwa H. Diffusion tensor imaging studies of mild traumatic brain injury: a meta-analysis. Journal of neurology, neurosurgery, and psychiatry. 2012;83(9):870–6. Epub 2012/07/17. doi: 10.1136/jnnp-2012-302742 ; PubMed Central PMCID: PMC3415311. 22797288 10.1136/jnnp-2012-302742 PMC3415311

[10] Gardner A, Kay-Lambkin F, Stanwell P, Donnelly J, Williams WH, Hiles A, et al A systematic review of diffusion tensor imaging findings in sports-related concussion. Journal of neurotrauma. 2012;29(16):2521–38. Epub 2012/09/07. doi: 10.1089/neu.2012.2628 . 22950876 10.1089/neu.2012.2628

[11] Lipton ML, Kim N, Park YK, Hulkower MB, Gardin TM, Shifteh K, et al Robust detection of traumatic axonal injury in individual mild traumatic brain injury patients: intersubject variation, change over time and bidirectional changes in anisotropy. Brain imaging and behavior. 2012;6(2):329–42. Epub 2012/06/12. doi: 10.1007/s11682-012-9175-2 . 22684769 10.1007/s11682-012-9175-2

[12] Muller HP, Unrath A, Riecker A, Pinkhardt EH, Ludolph AC, Kassubek J. Intersubject variability in the analysis of diffusion tensor images at the group level: fractional anisotropy mapping and fiber tracking techniques. Magnetic resonance imaging. 2009;27(3):324–34. Epub 2008/08/15. doi: 10.1016/j.mri.2008.07.003 . 18701228 10.1016/j.mri.2008.07.003

[13] Rosenbaum SB, Lipton ML. Embracing chaos: the scope and importance of clinical and pathological heterogeneity in mTBI. Brain imaging and behavior. 2012;6(2):255–82. doi: 10.1007/s11682-012-9162-7 . 22549452 10.1007/s11682-012-9162-7

[14] Bennett RE, Mac Donald CL, Brody DL. Diffusion tensor imaging detects axonal injury in a mouse model of repetitive closed-skull traumatic brain injury. Neuroscience letters. 2012;513(2):160–5. Epub 2012/02/22. doi: 10.1016/j.neulet.2012.02.024 ; PubMed Central PMCID: PMC3319388. 22343314 10.1016/j.neulet.2012.02.024 PMC3319388

[15] Eierud C, Craddock RC, Fletcher S, Aulakh M, King-Casas B, Kuehl D, et al Neuroimaging after mild traumatic brain injury: Review and meta-analysis. NeuroImage: Clinical. 2014;4(0):283–94. http://dx.doi.org/10.1016/j.nicl.2013.12.009. 10.1016/j.nicl.2013.12.009 PMC4107372 25061565

[16] Hulkower MB, Poliak DB, Rosenbaum SB, Zimmerman ME, Lipton ML. A Decade of DTI in Traumatic Brain Injury: 10 Years and 100 Articles Later. AJNR American journal of neuroradiology. 2013 Epub 2013/01/12. doi: 10.3174/ajnr.A3395 . 23306011 10.3174/ajnr.A3395 PMC7964847

[17] Schatz P, Pardini JE, Lovell MR, Collins MW, Podell K. Sensitivity and specificity of the ImPACT Test Battery for concussion in athletes. Archives of clinical neuropsychology: the official journal of the National Academy of Neuropsychologists. 2006;21(1):91–9. doi: 10.1016/j.acn.2005.08.001 . 16143492 10.1016/j.acn.2005.08.001

[18] Harris PA, Taylor R, Thielke R, Payne J, Gonzalez N, Conde JG. Research electronic data capture (REDCap)—a metadata-driven methodology and workflow process for providing translational research informatics support. Journal of biomedical informatics. 2009;42(2):377–81. doi: 10.1016/j.jbi.2008.08.010 ; PubMed Central PMCID: PMC2700030. 18929686 10.1016/j.jbi.2008.08.010 PMC2700030

[19] Koehler R, Wilhelim EE, Shoulson I. Cognitive Rehabilitation Therapy for Traumatic Brain Injury. The National Academies Press Washington, DC 2011.

[20] Smith SM, Jenkinson M, Johansen-Berg H, Rueckert D, Nichols TE, Mackay CE, et al Tract-based spatial statistics: voxelwise analysis of multi-subject diffusion data. NeuroImage. 2006;31(4):1487–505. doi: 10.1016/j.neuroimage.2006.02.024 . 16624579 10.1016/j.neuroimage.2006.02.024

[21] Mori S, Crain BJ, Chacko VP, van Zijl PC. Three-dimensional tracking of axonal projections in the brain by magnetic resonance imaging. Annals of neurology. 1999;45(2):265–9. Epub 1999/02/16. . 9989633 10.1002/1531-8249(199902)45:2<265::aid-ana21>3.0.co;2-3

[22] Wakana S, Caprihan A, Panzenboeck MM, Fallon JH, Perry M, Gollub RL, et al Reproducibility of quantitative tractography methods applied to cerebral white matter. NeuroImage. 2007;36(3):630–44. Epub 2007/05/08. doi: 10.1016/j.neuroimage.2007.02.049 ; PubMed Central PMCID: PMC2350213. 17481925 10.1016/j.neuroimage.2007.02.049 PMC2350213

[23] Hua K, Zhang J, Wakana S, Jiang H, Li X, Reich DS, et al Tract probability maps in stereotaxic spaces: analyses of white matter anatomy and tract-specific quantification. NeuroImage. 2008;39(1):336–47. Epub 2007/10/13. doi: 10.1016/j.neuroimage.2007.07.053 ; PubMed Central PMCID: PMC2724595. 17931890 10.1016/j.neuroimage.2007.07.053 PMC2724595

[24] Herrera JJ, Bockhorst K, Kondraganti S, Stertz L, Quevedo J, Narayana PA. Acute White Matter Tract Damage after Frontal Mild Traumatic Brain Injury. Journal of neurotrauma. 2016 doi: 10.1089/neu.2016.4407 . 27138134 10.1089/neu.2016.4407 PMC5220577

[25] Jiang Q, Qu C, Chopp M, Ding GL, Davarani SP, Helpern JA, et al MRI evaluation of axonal reorganization after bone marrow stromal cell treatment of traumatic brain injury. NMR in biomedicine. 2011;24(9):1119–28. doi: 10.1002/nbm.1667 ; PubMed Central PMCID: PMC3381889. 21432927 10.1002/nbm.1667 PMC3381889

[26] Ljungqvist J, Nilsson D, Ljungberg M, Sorbo A, Esbjornsson E, Eriksson-Ritzen C, et al Longitudinal study of the diffusion tensor imaging properties of the corpus callosum in acute and chronic diffuse axonal injury. Brain injury: [BI]. 2011;25(4):370–8. Epub 2011/03/02. doi: 10.3109/02699052.2011.558038 . 21355671 10.3109/02699052.2011.558038

[27] Tu TW, Williams RA, Lescher JD, Jikaria N, Turtzo LC, Frank JA. Radiological-pathological correlation of diffusion tensor and magnetization transfer imaging in a closed head traumatic brain injury model. Annals of neurology. 2016;79(6):907–20. doi: 10.1002/ana.24641 ; PubMed Central PMCID: PMC4887193. 27230970 10.1002/ana.24641 PMC4887193

[28] Li S, Sun Y, Shan D, Feng B, Xing J, Duan Y, et al Temporal profiles of axonal injury following impact acceleration traumatic brain injury in rats—a comparative study with diffusion tensor imaging and morphological analysis. International journal of legal medicine. 2013;127(1):159–67. doi: 10.1007/s00414-012-0712-8 . 22573358 10.1007/s00414-012-0712-8

[29] Budde MD, Janes L, Gold E, Turtzo LC, Frank JA. The contribution of gliosis to diffusion tensor anisotropy and tractography following traumatic brain injury: validation in the rat using Fourier analysis of stained tissue sections. Brain: a journal of neurology. 2011;134(Pt 8):2248–60. doi: 10.1093/brain/awr161 ; PubMed Central PMCID: PMC3155707. 21764818 10.1093/brain/awr161 PMC3155707

[30] Harris NG, Verley DR, Gutman BA, Sutton RL. Bi-directional changes in fractional anisotropy after experiment TBI: Disorganization and reorganization? NeuroImage. 2016;133:129–43. doi: 10.1016/j.neuroimage.2016.03.012 ; PubMed Central PMCID: PMC4889542. 26975556 10.1016/j.neuroimage.2016.03.012 PMC4889542

[31] Mayer AR, Mannell MV, Ling J, Gasparovic C, Yeo RA. Functional connectivity in mild traumatic brain injury. Human brain mapping. 2011;32(11):1825–35. doi: 10.1002/hbm.21151 ; PubMed Central PMCID: PMC3204375. 21259381 10.1002/hbm.21151 PMC3204375

[32] Schmidt M, Lipson H. Eureqa (Version 0.98 beta), Available from www.nutonian.com. 2013.

[33] Schmidt M, Lipson H. Distilling free-form natural laws from experimental data. Science. 2009;324(5923):81–5. Epub 2009/04/04. doi: 10.1126/science.1165893 . 19342586 10.1126/science.1165893

[34] Allgaier N, Tobias Banaschewski, Gareth Barker, Arun LW Bokde, Josh C. Bongard, Uli Bromberg, Christian Büchel et al. Nonlinear functional mapping of the human brain. 2015.

[35] Bendlin BB, Ries ML, Lazar M, Alexander AL, Dempsey RJ, Rowley HA, et al Longitudinal changes in patients with traumatic brain injury assessed with diffusion-tensor and volumetric imaging. NeuroImage. 2008;42(2):503–14. Epub 2008/06/17. doi: 10.1016/j.neuroimage.2008.04.254 ; PubMed Central PMCID: PMC2613482. 18556217 10.1016/j.neuroimage.2008.04.254 PMC2613482

[36] White T, Schmidt M, Karatekin C. White matter 'potholes' in early-onset schizophrenia: a new approach to evaluate white matter microstructure using diffusion tensor imaging. Psychiatry research. 2009;174(2):110–5. Epub 2009/10/27. doi: 10.1016/j.pscychresns.2009.04.014 ; PubMed Central PMCID: PMC2783844. 19853414 10.1016/j.pscychresns.2009.04.014 PMC2783844

[37] Davenport ND, Lim KO, Armstrong MT, Sponheim SR. Diffuse and spatially variable white matter disruptions are associated with blast-related mild traumatic brain injury. NeuroImage. 2012;59(3):2017–24. Epub 2011/11/02. doi: 10.1016/j.neuroimage.2011.10.050 . 22040736 10.1016/j.neuroimage.2011.10.050

[38] Jorge RE, Acion L, White T, Tordesillas-Gutierrez D, Pierson R, Crespo-Facorro B, et al White Matter Abnormalities in Veterans With Mild Traumatic Brain Injury. Am J Psychiat. 2012;169(12):1284–91. doi: 10.1176/appi.ajp.2012.12050600 . 23212059 10.1176/appi.ajp.2012.12050600 PMC4030599

[39] Ling JM, Pena A, Yeo RA, Merideth FL, Klimaj S, Gasparovic C, et al Biomarkers of increased diffusion anisotropy in semi-acute mild traumatic brain injury: a longitudinal perspective. Brain: a journal of neurology. 2012;135(Pt 4):1281–92. Epub 2012/04/17. doi: 10.1093/brain/aws073 ; PubMed Central PMCID: PMC3326260. 22505633 10.1093/brain/aws073 PMC3326260

[40] Mayer AR, Ling JM, Yang Z, Pena A, Yeo RA, Klimaj S. Diffusion abnormalities in pediatric mild traumatic brain injury. The Journal of neuroscience: the official journal of the Society for Neuroscience. 2012;32(50):17961–9. Epub 2012/12/15. doi: 10.1523/JNEUROSCI.3379-12.2012 . 23238712 10.1523/JNEUROSCI.3379-12.2012 PMC6621719

[41] Watts R, Thomas A, Filippi CG, Nickerson JP, Freeman K. Potholes and molehills: bias in the diagnostic performance of diffusion-tensor imaging in concussion. Radiology. 2014;272(1):217–23. doi: 10.1148/radiol.14131856 . 24635677 10.1148/radiol.14131856 PMC4263643

[42] Koerte IK, Kaufmann D, Hartl E, Bouix S, Pasternak O, Kubicki M, et al A prospective study of physician-observed concussion during a varsity university hockey season: white matter integrity in ice hockey players. Part 3 of 4. Neurosurgical focus. 2012;33(6):E3: 1–7. Epub 2012/12/04. doi: 10.3171/2012.10.FOCUS12303 . 23199426 10.3171/2012.10.FOCUS12303 PMC5687247

[43] Inglese M, Makani S, Johnson G, Cohen BA, Silver JA, Gonen O, et al Diffuse axonal injury in mild traumatic brain injury: a diffusion tensor imaging study. Journal of neurosurgery. 2005;103(2):298–303. doi: 10.3171/jns.2005.103.2.0298 . 16175860 10.3171/jns.2005.103.2.0298

[44] Assaf Y, Basser PJ. Composite hindered and restricted model of diffusion (CHARMED) MR imaging of the human brain. NeuroImage. 2005;27(1):48–58. Epub 2005/06/28. S1053-8119(05)00225-9 [pii] doi: 10.1016/j.neuroimage.2005.03.042 . 15979342 10.1016/j.neuroimage.2005.03.042

[45] Heiervang E, Behrens TE, Mackay CE, Robson MD, Johansen-Berg H. Between session reproducibility and between subject variability of diffusion MR and tractography measures. NeuroImage. 2006;33(3):867–77. doi: 10.1016/j.neuroimage.2006.07.037 . 17000119 10.1016/j.neuroimage.2006.07.037

[46] Setsompop K, Cohen-Adad J, Gagoski BA, Raij T, Yendiki A, Keil B, et al Improving diffusion MRI using simultaneous multi-slice echo planar imaging. NeuroImage. 2012;63(1):569–80. doi: 10.1016/j.neuroimage.2012.06.033 ; PubMed Central PMCID: PMC3429710. 22732564 10.1016/j.neuroimage.2012.06.033 PMC3429710

