# Supplementary figures and images for: Dynamic changes in diffusion measures improve sensitivity in identifying patients with mild traumatic brain injury
Source: PLoS One. 2017 Jun 12;12(6):e0178360. doi: 10.1371/journal.pone.0178360 (PMC5467843; doi:10.1371/journal.pone.0178360)

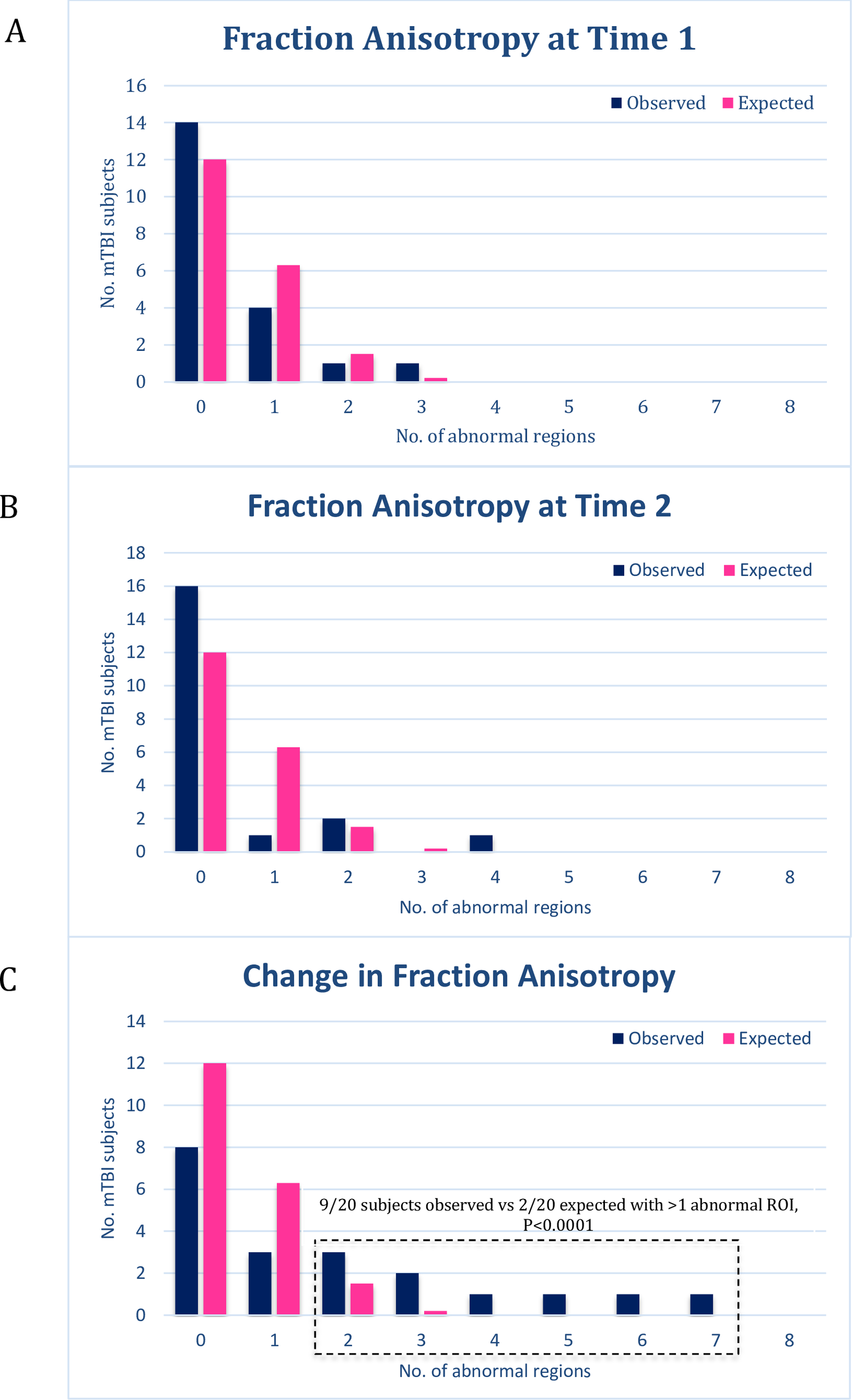

Supplement: S1 Fig — (A) Time 1, (B) Time 2 and (C) Change over time. (TIF) [file pone.0178360.s001.tif]

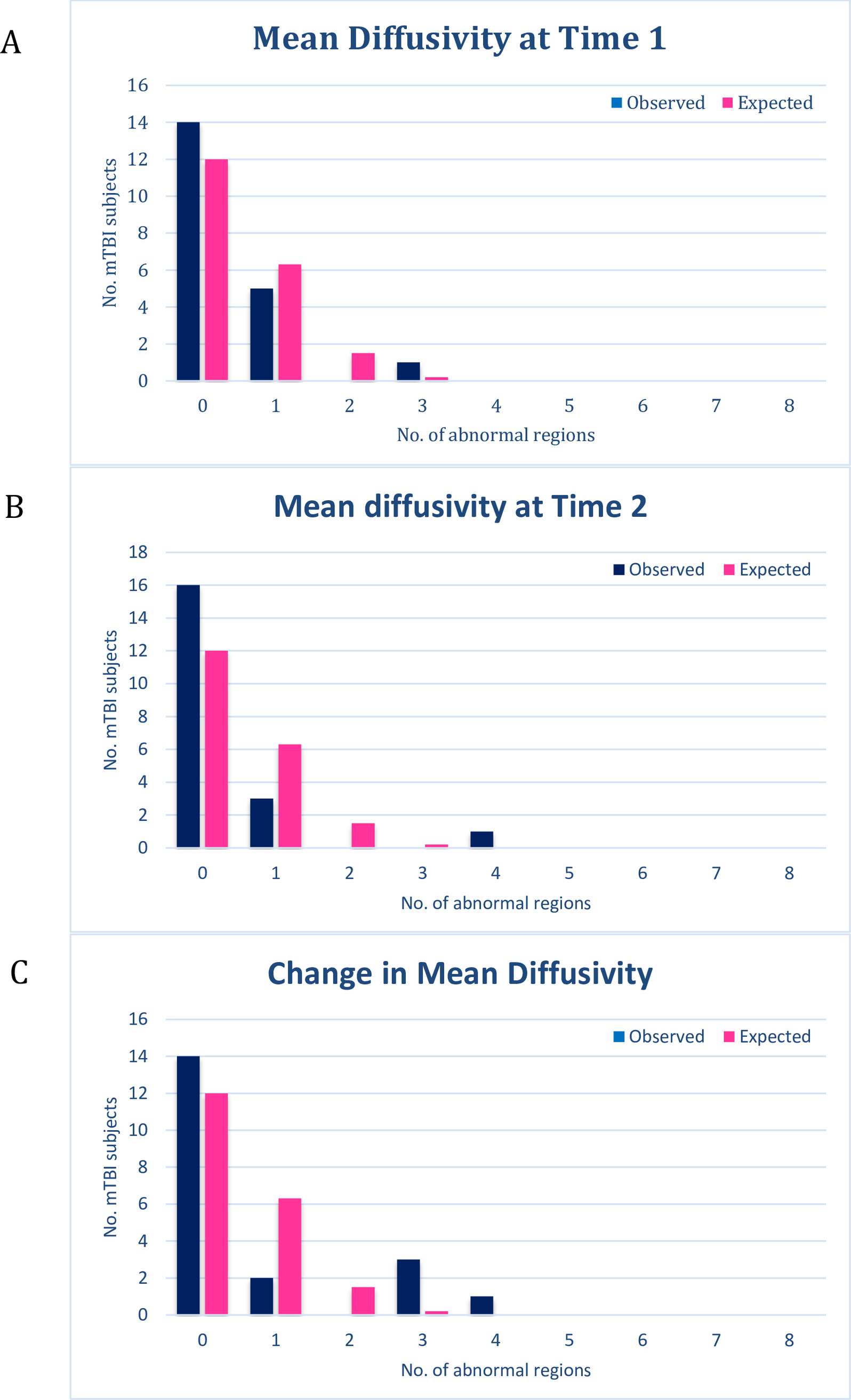

Supplement: S2 Fig — (A) Time 1, (B) Time 2 and (C) Change over time. (TIF) [file pone.0178360.s002.tif]

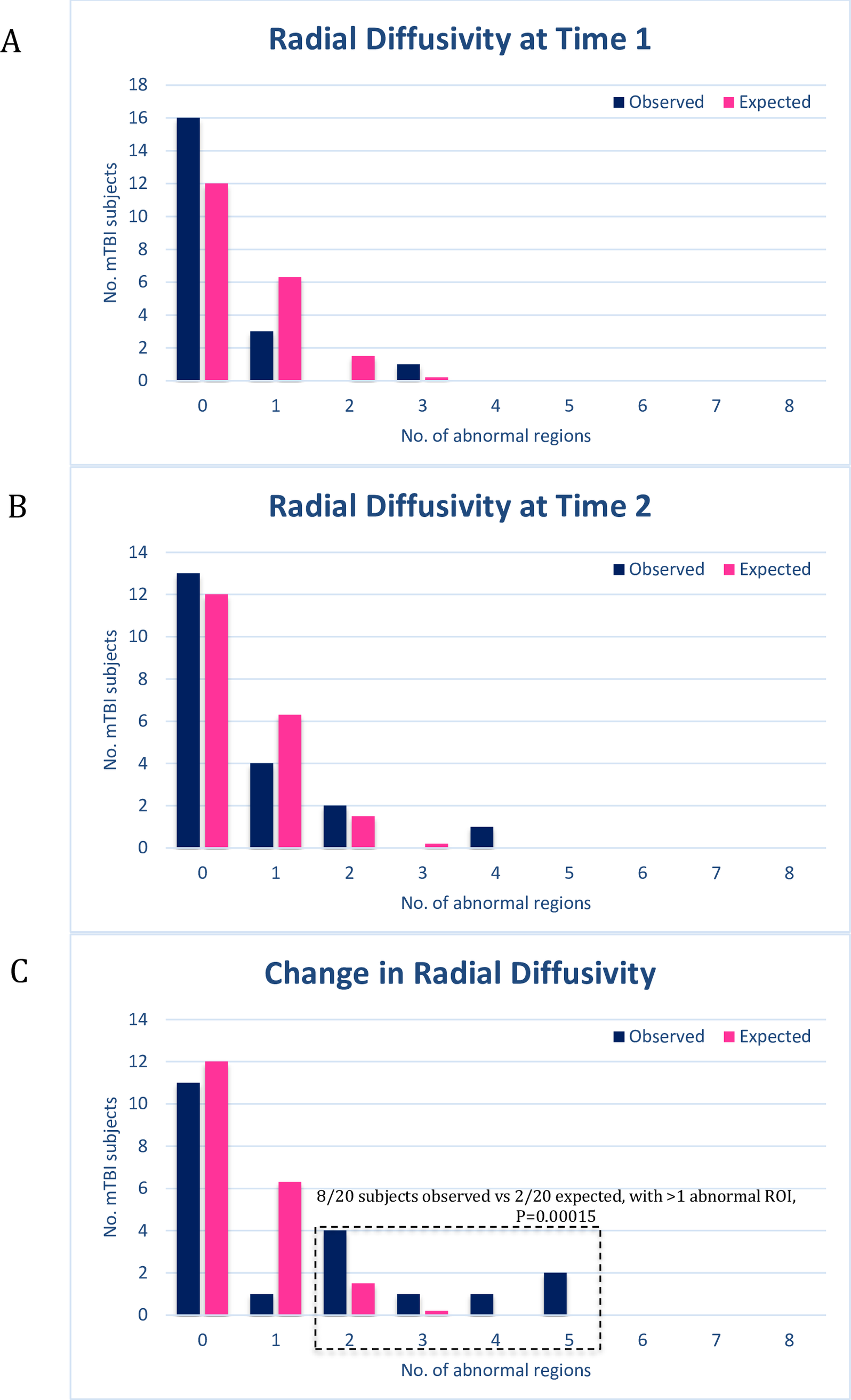

Supplement: S3 Fig — (A) Time 1, (B) Time 2 and (C) Change over time. (TIF) [file pone.0178360.s003.tif]

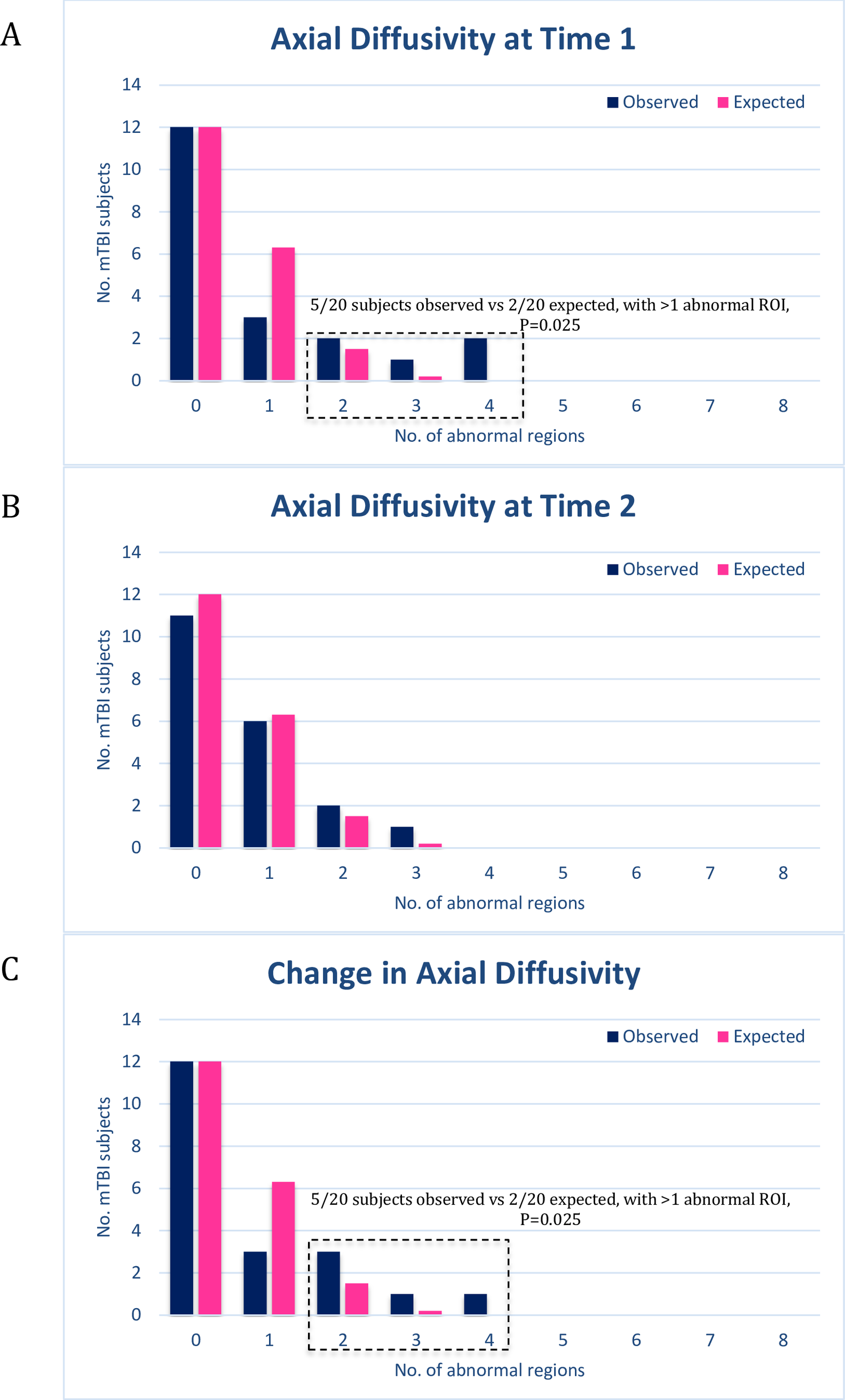

Supplement: S4 Fig — (A) Time 1, (B) Time 2 and (C) Change over time -4 *for S1–S4 Figs. Number of abnormal regions of interest in mild traumatic brain injury (mTBI) subjects. Abnormal regions are defined as having DTI metrics more than 2 standard deviations above or below the mean for the control group. Blue bars indicate the number of mTBI subjects with a given number of abnormal regions. Red bars indicate the number of subjects that would be expected by chance, based on a binomial distribution with n = 11 regions, p = 0.0455. Regions are assumed to be independent (8). Dashed boxes indicates metrics in which the number of mTBI subjects with more than one abnormal region is significantly different to that expected by chance (binomial distribution, n = 20 subjects, p = 0.0867). (TIF) [file pone.0178360.s004.tif]
