# Supplementary material for: Dynamic changes in diffusion measures improve sensitivity in identifying patients with mild traumatic brain injury
Source: PLoS One. 2017 Jun 12;12(6):e0178360. doi: 10.1371/journal.pone.0178360 (PMC5467843; doi:10.1371/journal.pone.0178360)
Supplement: S1 Table — (DOCX) [file pone.0178360.s005.docx]

* All values for axial diffusivity are x10^-3^
