# Supplementary material for: Dynamic changes in diffusion measures improve sensitivity in identifying patients with mild traumatic brain injury
Source: PLoS One. 2017 Jun 12;12(6):e0178360. doi: 10.1371/journal.pone.0178360 (PMC5467843; doi:10.1371/journal.pone.0178360)
Supplement: S5 Table — (DOCX) [file pone.0178360.s009.docx]

|  | Number | Percentage |
| --- | --- | --- |
| Motor vehicle crash | 1 | 5% |
| Sports injury | 12 | 60% |
| Assault/blunt trauma | 4 | 20% |
| Other fall | 3 | 15% |
