# Supplementary material for: Dynamic changes in diffusion measures improve sensitivity in identifying patients with mild traumatic brain injury
Source: PLoS One. 2017 Jun 12;12(6):e0178360. doi: 10.1371/journal.pone.0178360 (PMC5467843; doi:10.1371/journal.pone.0178360)
Supplement: S6 Table — Binary masks defining each region of interest were transformed into the individual subject space using nearest neighbor resampling. This prevented any possible effect of smoothing of the original data, although the mask definitions are likely to be somewhat less accurate. For control subjects, the standard deviation of FA values for each ROI at time point 1 and 2, along with the standard deviation of change within subjects across the two time points is shown. (DOCX) [file pone.0178360.s010.docx]

Corpus callosum (CC), posterior limbs of the internal capsule (PLIC), uncinate fasciculus (UF), corona radiata (CR) and corticospinal tract (CST).
