# Supplementary material for: Dynamic changes in diffusion measures improve sensitivity in identifying patients with mild traumatic brain injury
Source: PLoS One. 2017 Jun 12;12(6):e0178360. doi: 10.1371/journal.pone.0178360 (PMC5467843; doi:10.1371/journal.pone.0178360)
Supplement: S1 Methods — (DOCX) [file pone.0178360.s011.docx]

Supplementary Methods

**Imaging post-processing**:

1. Performed DICOM to NiFTI conversion using MRI_convert (<https://surfer.nmr.mgh.harvard.edu/fswiki/mri_convert>)
2. Eddy_correct (<http://fsl.fmrib.ox.ac.uk/fsl/fslwiki/EDDY>)
3. DTIfit (<http://fsl.fmrib.ox.ac.uk/fsl/fslwiki/FDT/UserGuide#DTIFIT>)
4. tbss_1_preproc using FMRIB_FA template (<http://fsl.fmrib.ox.ac.uk/fsl/fslwiki/TBSS/UserGuide>)
5. tbss_2_reg using FMRIB_FA template (<http://fsl.fmrib.ox.ac.uk/fsl/fslwiki/TBSS/UserGuide>)
6. tbss_3_postreg using FMRIB_FA template (<http://fsl.fmrib.ox.ac.uk/fsl/fslwiki/TBSS/UserGuide>)
7. fslstats using JHU atlas provided with fsl (<http://fsl.fmrib.ox.ac.uk/fsl/fslwiki/Atlases>)
